# Supplementary material for: Marine deforestation leads to widespread loss of ecosystem function
Source: PLoS One. 2020 Mar 4;15(3):e0226173. doi: 10.1371/journal.pone.0226173 (PMC7055868; doi:10.1371/journal.pone.0226173)
Supplement: Attachment — Submitted filename: Editor comments_answered.docx [file pone.0226173.s001.docx]

Maura (Gee) Geraldine Chapman, PhD DSc
Academic Editor
PLOS ONE
Dear Dr. Chapman

Thank you for the thoughtful review of our manuscript “Marine deforestation leads to widespread loss of ecosystem function”. We greatly appreciate the detailed comments the reviewers provided. We have carefully gone through the comments and have adopted nearly all of them, which we believe has greatly improved this manuscript. In our revised manuscript, we have gone thorough great effort to reanalyze much of the data, and have explored the complexities of our production patterns among islands and years. This has very much added to the story, and has revealed that deforestation does indeed result in geographically complex patterns of productivity. However, even with this, general patterns remain clear. We have explored these complexities and generalities, adding two new tables that highlight these issues. We have strengthened our arguments, adding new citations that provide historical context. We believe this is a much stronger manuscript as a result. In the pages below, we detail how we addressed each comment made by the reviewers (see type in red font). In the end, we have adopted nearly every comment as suggested. The few comments that we did not address, we have added language clarifying things, which we hope should satisfy the reviewers concerns.

We would like our paper considered for the Biodiversity Conservation Call for Papers, as suggested in the letter to us. This revised paper has 8 figures, 5 tables, and 62 citations. No data are being considered for publication elsewhere. We have included a marked copy of the paper with track changes visible, and a clean copy with them accepted. We also upload the figures and tables as separate documents.

Thank you for the effort and consideration.

Matt Edwards. Lead Author

San Diego State University

1. Please ensure that your manuscript meets PLOS ONE's style requirements, including those for file naming. The PLOS ONE style templates can be found at <http://www.plosone.org/attachments/PLOSOne_formatting_sample_main_body.pdf> and <http://www.plosone.org/attachments/PLOSOne_formatting_sample_title_authors_affiliations.pdf>

Done. We have reviewed the instructions and changed the format of our figures and tables, and the way they are cited.

2. In your Methods section, please provide additional information regarding the permits you obtained for the work. Please ensure you have included the full name of the authority that approved the field site access and, if no permits were required, a brief statement explaining why.

Done. We had added the following statement to the end of the Methods section, “Ethical Approval: All procedures performed in studies involving fishes were in accordance with the ethical standards of the institution or practice at which the studies were conducted (University of Alaska Fairbanks Institutional Animal Care and Use Committee; Permit Number: 899401-4).”

3. Our internal editors have looked over your manuscript and determined that it is within the scope of our Biodiversity Conservation Call for Papers. This collection of papers is headed by a team of Guest Editors for PLOS ONE (<https://collections.plos.org/s/biodiversity>). The Collection will encompass a diverse range of research articles on biodiversity conservation, including the impacts of deforestation and habitat loss. Additional information can be found on our announcement page: <https://collections.plos.org/s/biodiversity>
If you would like your manuscript to be considered for this collection, please let us know in your cover letter and we will ensure that your paper is treated as if you were responding to this call. If you would prefer to remove your manuscript from collection consideration, please specify this in the cover letter.

 Yes thank you. We would like this considered.

4. Thank you for stating the following in the Acknowledgments Section of your manuscript:

"This research was funded by grants from the National Science Foundation (OCE1435194) to MSE and BK, and the National Research Foundation (NRF-2018R1C1B6008523 and NRF-2015R1C1A1A01054831) to JHK."

We note that you have provided funding information that is not currently declared in your Funding Statement. However, funding information should not appear in the Acknowledgments section or other areas of your manuscript. We will only publish funding information present in the Funding Statement section of the online submission form.

Please remove any funding-related text from the manuscript and let us know how you would like to update your Funding Statement. Currently, your Funding Statement reads as follows:

"NO - The funders had no role in study design, data collection and analysis, decision to publish, or preparation of the manuscript."

Done! We have removed the statement and have inserted it into the funding statement. “Funding: This research was funded by grants from the National Science Foundation (OCE1435194) to MSE and BK, and the National Research Foundation (NRF-2018R1C1B6008523 and NRF-2015R1C1A1A01054831) to JHK.

5. We note that Figure #2 in your submission contains map images which may be copyrighted. All PLOS content is published under the Creative Commons Attribution License (CC BY 4.0), which means that the manuscript, images, and Supporting Information files will be freely available online, and any third party is permitted to access, download, copy, distribute, and use these materials in any way, even commercially, with proper attribution. For these reasons, we cannot publish previously copyrighted maps or satellite images created using proprietary data, such as Google software (Google Maps, Street View, and Earth). For more information, see our copyright guidelines: <http://journals.plos.org/plosone/s/licenses-and-copyright>.

We require you to either (a) present written permission from the copyright holder to publish these figures specifically under the CC BY 4.0 license, or (b) remove the figures from your submission:

We created our own figure map using published data. Specifically, shoreline data were obtained from the Global Self-Consistent Hierarchical High-resolution Shoreline dataset version 2.3.4 ([www.soest.hawaii.edu/wessel/gshhg/)](http://www.soest.hawaii.edu/wessel/gshhg/)) (Wessel & Smith 1996). The site where we obtained the data states "GSHHG is released under the GNU Lesser General Public license, and is developed and maintained by Dr. Paul Wessel, SOEST, University of Hawai'i, and Dr. Walter H. F. Smith, NOAA Laboratory for Satellite Altimetry". We have included the following text in the Figure caption. “Shoreline data was obtained from the Global Self-Consistent Hierarchical High-resolution Shoreline (GSHHG) dataset version 2.3.4 ([www.soest.hawaii.edu/wessel/gshhg/)](http://www.soest.hawaii.edu/wessel/gshhg/)) (Wessel and Smith 1996).”

We have also added the following citation for this.

Wessel P, Smith WHF. 1996 A global, selfconsistent, hierarchical, high-resolution shoreline
database. J. Geophys. Res. Solid Earth 101(B4), 8741– 8743. (doi:10.1029/96JB00104)

6. Please upload a copy of Supporting Information Table S1 which you refer to in your text on page 23.

This reference was cited incorrectly. It should have read Table 1 (not S1). This table is is now lncluded as Table 4.

**Comments to the Author**


Have the authors made all data underlying the findings in their manuscript fully available?

The [PLOS Data policy](http://www.plosone.org/static/policies.action#sharing) requires authors to make all data underlying the findings described in their manuscript fully available without restriction, with rare exception (please refer to the Data Availability Statement in the manuscript PDF file). The data should be provided as part of the manuscript or its supporting information, or deposited to a public repository. For example, in addition to summary statistics, the data points behind means, medians and variance measures should be available. If there are restrictions on publicly sharing data—e.g. participant privacy or use of data from a third party—those must be specified.

We have deposited our data in our BCO-DMO data page with NSF. We have inserted the following statement . **Data and materials availability**: Data are available on our NSF bco-dmo data page at <https://www.bco-dmo.org/dataset/755658>

 
Reviewer #1: The authors report on productivity estimates in the nearshore of the Aleutians, studying a community that has likely changed as a result of orca whale predation on sea otters, thus leading to high urchin densities in some areas. The authors use chambers in situ to measure productivity across habitat types that differ: kelp-dominated versus crustose coralline-dominated. The measurements they have made in the field with these chambers are valuable and include the whole in situ community. The effort to do this was significant. I thus think this is a very worthwhile study, though I have several comments to increase the clarity of the presentation and interpretation.

First, I was surprised not to see any mention of seastar die-off as a driver of high urchin abundances. Are the findings of Burt et al. 2018 (PNAS) in Canada irrelevant here? Are there any data for seastar die-offs?

We appreciate the link to seastar die-offs but this ecosystem shift began years prior to the seastar die-offs. Estes and colleagues reported the first signs of this in the 1970’s and we have followed the collapse of the kelp forests since the early 1990’s. Thus, while seastar die-offs have likely been a major force in sea urchin population expansions along other areas of the west coast of North America, our kelp forest collapses began long before the seastar mortality and were thus not a likely contributor. However, given the large focus on this phenomenon in recent years, we believe this comment is worthy of addressing. Thus, we have added language to let the reader know that seastar die-offs were not likely causative in the kelp collapse in the Aleutian Islands.

That being said…the reviewer brings up a salient comment about the role of other predators that may be important in the face of sea otter declines. We have thus referenced papers by Hewson et al.(2014), Menge et al. (2016) and Burt et al (2018) in our discussion where we discuss the potential for other predators to exert top-down influence on urchin populations. Below is the language we inserted.

“One potential contributing factor for this may lie in the low abundance of other urchin predators such as sea stars (Burt et al 2018), which exhibited widespread mortality throughout the northeastern Pacific beginning in 2013 due to the sea star-associated densovirus disease (Hewson et al 2014, Menge et al. 2016 Burt et al 2018.). While these populations have shown some signs of recovery in parts of their ranges, they remain in low abundance in the Aleutian Archipelago relative to their historical condition (MSE and BK, personal observation). Until predation on the urchins recovers, benthic biodiversity, *GPP* and *Re* will likely remain lower in areas of kelp forest loss because the high abundance of urchins limits regrowth of macroalgae and maintains the urchin barrens.”

Second, and importantly, parts of the Discussion contradict the Results. For example, at L 230 it is stated that GPP and Re will remain lower in areas of kelp forest loss. Yet, Figs show Re is higher. Again on L 239, L252 In fact, the Figs show increased GPP and decreased Re in kelp forests. Is there a mistake on your figs or in the text explanation? Or did I miss something?

Nothing was missed. Re is indeed lower, on average, in areas of kelp loss. We recognize that the confusion here comes from the fact that the y-axis is presented in negative numbers (as oxygen is consumed not produced via respiration) and thus data that are elevated in the figure are actually closer to zero (less negative). Given this confusion here, we believe the pattern is clearer if we revise the figure to alter the sign and present respiration in positive numbers. We believe this has cleared thigs up.

Even though the data were analyzed with a mixed effects model using island as a random effect, the fact that islands differ make this difficult to interpret. In general, the explanations that the reader gets in the Results and Discussion do not match the complexity of the data.

We agree. First, we have redone our analyses to include Year as a factor. Second, because there was a Habitat*Island(Year) interaction for Each if the metrics we analyzed (see revised Table 2), we have dropped the post hoc tests of habitat, which we again agree was an oversimplification. Rather than running 27 post hoc tests for the three habitat comparisons at each of the nine islands (which would likely result in some erroneous comparisons due to low replication, n = 3), we instead present the production values and errors for each metric (see Table 3), and changes in each production metric at each island as the habitat changes from Kelp to Transition, from Transition to Urchin Barren, and the total change due to deforestation as the habitat changes from Kelp to Urchin Barren (see Table 4). Thus, while the overall (average) effects were clear when examined across all islands, these tables show that the effects were variable among islands visited in the two study years, and this is supported by the Habitat&*Island(Year) interaction. We have explored this in greater detail in both the results and discussion, and has become a central theme of the results.

L46 unnecessary text: “Nowhere may this be as dramatic as..”

We have removed this text.

L112 canopy algae also host a diversity of microbes directly (Michelou 2013 PlosOne, Weigel and Pfister 2019 Front in Microb).

Thank you for these references. We have added them to the text.

L121 “Understanding the balance…to NEP is a key aspect to understanding how habitat-driven ecosystem change occurs. We test the relationship among GPP, Re, NEP in the Aleutian Archipelago where recent widespread …” I though this section needed a much more direct statement.

Agreed. We have revised this to read, “Thus, understanding how *GPP, Re* and *NEP* change with kelp loss can be instrumental in discerning the broader impacts of deforestation on ecosystem health and productivity.”

L146 Benthic irradiances as photosynt active rad (PAR)…

We have changed this to read: Benthic irradiances, measured as *photosynthetically active radiation* (*PAR*),

L196 and in discussion: perhaps replace ‘widespread kelp deforestation’ with ‘large-scale habitat change’. The primary producers have changed and that is your point

We have done this in some places (e.g.. Line 196) where we believe it reads well. We have left deforestation in place in other places as we feel it more clearly defines the type of habitat change we are addressing.

L263, 264 – I recall coral reef in situ chambers by Williams and Carpenter 1998 that came much before the cited papers.

Agreed. We have added this paper to the references..

Table 2 – I’m glad the authors used island as a random factor

Thank you. This was necessary given the sampling design.

Table 4 – what do interactions tell us? Were they discussed? Are these interactions important in the confusing reporting of GPP and Re trends?

Agreed. As detailed in the response above, we have reanalyzed our data to include years, and the habitat*island(year) interaction. This showed that the patterns for each metric were not consistent across the islands. We explore this in greater detail in Tables 3 and especially Table 4, and in the results and discussion.

Reviewer #2: Edwards et al. provide a novel approach to quantifying the effects of trophic downgrading on kelp forest ecosystems. By empirically quantifying ecosystem rates across high latitude temperate reefs varying in macroalgal biomass, this manuscript offers a unique and important contribution to our understating of kelp forest dynamics globally. The strength of this paper lies in the collection of these empirical rates. Moreover, the monumental field effort to acquire these empirical rate data across 1000km is remarkable and these hard earned and unique data should be reported in the literature.

There are also several key weaknesses to this paper that the authors could address by fine tuning the writing, hypotheses and quantitative analyses. First, the analyses and figures do not tell the story the manuscript’s introduction and hypotheses aim to set up. Specifically, the alternative hypotheses presented relate GPP, Re and NEP to variation in primary producer biomass driven by the deforestation of kelp by sea urchins (Lines 114- 123, 155-156).

Agreed. We have changed this to reflect habitat type rather than biomass. We do this because 1) we do not have biomass measurements for all islands, and 2) we chose our habitat a priori to reflect non-overlapping habitats and thus do not allow biomass to vary naturally. This was necessary because the habitats of the Aleutians really occur as one of three states with very little overlap. Thus, while the reviewer makes a salient point, we do not have complete data for this analysis, and the data that we do have would not be naturally varying unbiased estimates of biomass (as they were chosen to represent one of three habitat types). Instead, we again link the differences to these three habitat types as a categorical variable. We have inserted language to reflect this.

Moreover, the authors suggest that ‘complex patterns’ of these rates may be driven by variation in macroalgal biomass, irradiance and secondary producer biomass and biodiversity. However, the quantitative models and the graphs themselves never assess these relationships and plausible causal mechanisms. Instead, habitat was used as a categorial variable and proxy for primary producer biomass. An alternative approach would be to run a set of linear models with primary producer biomass as a covariate, along with other important covariates implicated by the authors but not tested (Lines 116-121). The authors could also choose to keep their analyses as is yet better articulate and illustrate their hypotheses under various categorical scenarios of biomass, irradiance and secondary production. When alternative hypotheses and the variables they invoke match the empirical data collected, tested and presented, it makes it much easier for future readers to understand the evidence in support of alternative hypotheses.

While we again agree this would be an interesting analysis, this is not how we collected our data. We do not have orthogonal sets of PAR, biomass data to go with the production estimates, as we did not sample biomass in 2016. Also, the biomass would not be objectively chosen, as we selected sites and placements a priori based on the biomass present reflecting the habitat in question. These habitats are categorical and occur as one of three non-overlapping states. Thus, we revise our discussion to reflect the non-overlapping conditions of the three habitat types, and have inserted language to clarify this.

Second, as the authors know well, factors beyond herbivory are well known to influence kelp forest structure and function. The manuscript would be stronger if these additional drivers of change were acknowledged in the text and addressed in the quantitative analysis. Specifically, the analyses considered ‘island’ as a random factor and ‘habitat’ as a fixed effect. Yet, across 1000km, surely these islands, and the habitats nested within them, varied in some biotic and abiotic conditions other than herbivory. This makes it difficult to treat islands as quantitative ‘replicates’. Did islands and habitats where the rates were collected also vary in wave exposure, depth, nutrients and sea water temperature? Might these factors have influenced the ecosystem rates measured? I would think so to some degree over this massive geographical area. Perhaps the authors chose sites to reduce these variables. If so, this should be clearly stated. At most this could be tested, at least this should be acknowledged.

Agreed. We have acknowledged this in the site descriptions. We have revised this to say “These islands span more than 1000 km and therefore experience differences in temperature, salinity, wave exposure and other biotic factors (see new citation by Bland et al. 2019). Consequently, all cBITs deployments were done in the summer (i.e. July) of each year, in similar depths (i.e. 6-8 m), and under similar wave exposures (i.e. protected from ocean swells) in order to standardize factors that could affect productivity measurements. The three habitat types were selected based on non-overlapping community assemblages (i.e. kelp forests were chosen based on abundant *E. fistulosa* and dense assemblages of understory macroalgae; transition habitats were chosen based on abundant *E. fistulosa*, little-to-no understory macroalgae, and high abundances of urchins; urchin barrens were chosen based on no *E. fistulosa*, little-to-no understory macroalgae and abundant urchins). These were then grouped in each island to reduce the effects of working across such a large geographic area.”

Third, the quantitative approach seems a bit awkward given the hypotheses tested. Why were permutation tests done on transformed data when a simple generalized linear model with an appropriate link function be more parsimonious? There may be a good reason for this, it just wasn’t clear to me. It would be helpful if the authors justified their quantitative approach in the text.

Agreed. We redid these analyses as generalized mixed effects models, which told the same story. However, upon discussion, we realize that the results of these analyses are certainly not unbiased, but rather circular. We chose the placements of the chambers to reflect the habitats (which were non-overlapping in their assemblages). Thus, p-values would not be informative but rather would just acknowledge we placed our chambers in each habitat. We have dropped these analyses from the paper and instead cite the abundances of algae and invertebrates, and use this simply to show they reflect the habitats of choice. We have inserted language to reflect this.

Were the 3 replicates done in the same location within a habitat type nested within an Island?

Habitats were orthogonal with islands and years. We chose our three habitats based on the definitions discussed in the comment above, which represented three truly non-overlapping habitat types. We have clarified our placements and choice of habitats within each island in the methods section

Minor Points
Data collection was done in 2016 and 2017, but the authors do not specify which sites were sampled in what year. As temperature and other anomalies vary between years, did the authors test for an effect of year in their analysis?

First, we have added a column to Table 1 that states which islands were sampled in which years. Second, we reanalyzed our data to include year in the analyses. This was an oversight earlier. The new analyses have Habitat, Year, Island(Year), Habitat*Year, and Habitat*Island(Year), which we believe is the appropriate linear model for this data set. Also as discussed ablve, this identified a significant Habitat*Island(Year) interaction, which became a major part of the results, and is explored in Tables 3 and 4, and in the discussion.

The authors’ use of the term ‘ecosystem health’ is confusing because it obfuscates what is inferred from what was measured. To clarify their inferences, I recommend that the authors consider removing this nondescript term and instead use the word to describe the metric measured.

Agreed. We have removed this term throughout the manuscript.

Biodiversity is mentioned throughout the text but never quantified.

Agreed. We have changed this to reflect reduced macroalgal and sessile invertebrate abundances rather than biodiversity.

Nowhere in the manuscript is variation in sea otter abundance or occupation time reported and yet the inferences made about deforestation are all related to sea otter depletion.

We appreciate this comment, but the issue here is that the otters had disappeared from these islands years prior to our study and that the kelp forests had also disappeared (barrens formed) years before the study. This is how we motivate the study in the introduction. Our habitats were chosen a priori to reflect the results of kelp forests being absent and urchin barrens having been formed. This pattern along with otters being nearly absent are widespread throughout the island chain and the exact timing of when the otter disappeared does not seem to have created variation in the habitat types they truly are in one of three states (barrens, kelp, transition) and these are mutually exclusive. Thus, while the historical timing of when the otters disappeared years prior may vary among islands, all these islands had been otter-free (or nearly so) for some time and the condition of the habitats we identified was not dependent on this timing. We believe we make this clear in the introduction where we motivate the study

A substantial amount of inference appears in the results section (ex: lines 161-163, 166-169, 183-201). I would recommend keeping the inference to the discussion section. Moreover, some of the inferences (ex: lines 189-191) made could be tested with data (in this case PAR measurements).

Agreed. We have moved much of this inference to the discussion. However, in a couple places these single sentences directly link to the preceding passages where we present the results of our experiments.

There are inconsistencies in the definitions of NEP (lines 99-100 & 169-171) that make for a circular argument presented in the discussion (lines 174-177).

Agreed. We have revised Lines 174-177 to change “difference” to’ “range” and added “alone” after NEP.

To improve Fig 4B, consider graphing the encrusting invertebrates in a separate graph with a smaller y axis scale so readers can see the differences in inverts among kelp, transitions sites and urchin barrens. These are great data and I would love to see them better.

Agreed. We have graphed this figure into separate panels as suggested. This should better show differences among habitats for the less abundant taxa.

Fig 7 and 8: Consider joining them into one figure

agreed. We have done this.

Line 105: Two ‘indeeds’

This has been corrected

Line 119: Benthic irradiances not defined

We have defined it here. The passage now reads “…also results in increased benthic irradiances (measured as photosynthetically active radiation - *PAR*)

Line 130: Define what defines a transition site? Sea otter occupation? Kelp density?

Thank you. We have clarified this. The passage now reads: “…habitats that were in transition to becoming urchin barrens (i.e. they had lost all benthic macroalgae but still had abundant stands of the canopy-forming *Eualaria fistulosa*; Fig. 1)”

Line 294 – cBITs deployed for 24-36 hour periods. How does variation in deployment time affect results?

We have corrected this. Deployment times were 36 hours. The 24 hours was the original intent to capture a complete diurnal cycle but we expanded this to ensure we captured the complete cycle in the face of time needed to deploy and pick them up. The original writing of this passage was left over from when we wrote the proposal.

Line 169: ‘Lastly, the difference (i.e. range) between GPP and Re, which we believe to be a better measure of ecosystem function than NEP..’ but in lines 99-100 you write: ‘net ecosystem production (NEP),which is the difference between GPP and Re.’

Thank you. We agree this was confusing. We have removed the word “difference” and modified the statement to read “Lastly, the range between GPP and Re, which we believe to be a better measure of ecosystem function than NEP…”.

line 179: indicted should be indicated

We have made this change

Lines 238–240: Unclear. Does ecosystem health and function = biodiversity, macroalgal abundances, and primary productivity?

We agree this was confusing. We have altered the passage to clarify this. It now reads, “Certainly, benthic *GPP,* *Re* and the range between them are all greatest in the kelp forests where macroalgae, fish, invertebrate, and presumably microbial, communities are all most abundant. Deforestation then resulted in reductions in macroalgae, fish and invertebrate abundances, and corresponding decreases in primary productivity and respiration by the ecosystem. In contrast, macroalgae, fish and invertebrate abundances, primary productivity and respiration by the ecosystem were all similar in the urchin barrens and transition habitats, suggesting that the transition habitats have already suffered reduced ecosystem functioning.”

Methods: Please specify depth and season when cBITs were deployed as kelp growth, photoperiod, upwelling intensity, phytoplankton blooms, and wave exposure vary as a function of season, and all of these factors could influence GPP, Re, and NEP.

We have modified this passage to read “. All cBITs deployments were done in the summer (i.e. July) of each year, in similar depths (i.e. 6-8 m), and under similar wave exposures (i.e. protected from ocean swells) in order to standardize factors that could affect productivity measurements.”

Line 545 Table 1: It would be nice to see totals for number of CBITs deployed by habitat type. Could also add collection year/month as a column. This Table could be in an Appendix.

We agree. We have added the sample year as a new column to this table, and summed the number of deployments at each habitat and total collections made.

Reviewer #3: Review of PONE-D-19-32311 by Edwards et al

The authors present results from an interesting study examining changes in ecosystem functioning following a shift in Alaskan coastal habitats from kelp forests to urchin barrens. The study is well conceived and executed, the paper is well written and the data are analysed and interpreted appropriately. In fact, I really enjoyed reading the paper and think it will be very well received by the marine ecological community. It provides a neat and pertinent example of how structural shifts alter functioning and also raises some interesting points about the usefulness of GPP, NPP and Re measurements in coastal systems. I have some relatively minor concerns and suggestions for improvement that the authors should consider.

Line 66-67: On first reading I found the link between predators and forest-forming trees odd as predators don’t eat trees. I think either change ‘predators’ to ‘consumers’ or insert ‘directly or indirectly’ after ‘they result’

Agreed. We have changed predators to consumers.

Line 85: The collapse of sea otter populations in the region began a long time before the 1980s due to hunting otters for fur. I think it would be useful to add a little more historical context here for readers unfamiliar with the system. Maybe just clarify that this recent collapse of sea otter numbers comes after a historical collapse and recovery.

While hunting of otters for fur was certainly done during the previous century (driving the populations to near extinction), this hunting stopped more than a century ago when the species was protected, leading to rebounds throughout the Aleutian Archipelago. In reality, the otter populations had recovered to near carrying capacity throughout much of the island chain by 1980. Thus, the collapse of the populations beginning around 1980 that we discuss in this paper was not due to human hunting but rather is believed due to predation by Killer whales as reported by Estes et al. (1998). While we will agree to add verbiage to this historical collapse during the fur trade of the 19^th^ century if we are required to, we do not have historical data for the production or biomass metrics we studied and thus we believe that this may confound the current story. We therefore believe the case is stronger/clearer if we keep the inference as is.

Line 88: Somewhere in the introduction, maybe around here, it would be good to provide some details about the kelp forests themselves. I had to read to the discussion before the actual species of kelp was mentioned. Just briefly, what is the general structure of these forests? Does a single species dominate or are a few species important?

We have done this.

Line 91: I think the word ‘excellent’ is slightly subjective. Perhaps ‘useful’ or ‘valuable’ would be more scientific?

Agreed. We have changed this to valuable

Line 208: Similarly, replace ‘excellent’ with useful or pertinent
Agreed. We have changed this to pertinent

Line 210-215: I feel this section is a little unbalanced, as it implies that many (most?) kelp forests are in decline, whereas in fact most kelp ecosystems globally are stable and some are even expanding into polar regions. The paradigm that kelp forests are structured by trophic cascades is not true in many (most?) regions and the idea that they are in decline in most regions is also not supported by field observations. I think it should be made explicit that many kelp forests are intact, to provide balance for the statements focussing on kelp loss. For example, Krumhansl et al (2016 PNAS) show that kelps in 62 ecoregions studied were either stable or increasing and Smale (2019 New Phytologist) showed that kelp populations are expanding at high latitudes in response to warming. Finally, some of the references provided in the section don’t really support the statement being made. Specifically Pfister et al (ref 34) showed that kelp populations in the northeast Pacific were by and large stable, and declines were very localised. Similarly, Raybaud et al 2013 (ref 36) examined a single species and showed it has/will decline in France but this doesn’t really support ‘kelp forests’ or ‘Western Europe’. Again, the paper by Martinez et al (ref 39) used species distribution models to project future distributions of seaweeds in southern Australia, they did not show that they have exhibited declines. This may be better rephrased as ‘southwest Australia’ and supported by Wernberg et al 2013 Nat Clim Change or Wernberg et al 2016 Science which did show kelp decline in this region.

Agreed. We were trying to make this case too directly. We have greatly revised this passage to include Krumhensl et als. work suggesting stability in many ecoregions, Smale’s work showing increases in some high latitudes, and have added Wernberg’s two citations. We have also added work by Reed et al, Pfoster et al, showing local stability of some kelp forests and added a review by Smale. We have removed references 36 and 39. We think the passage now better reflects real world declines, stability and increases in kelp to show that kelp deforestation is not universal but that it has occurred in some regions.

Line 306: Which time of year were the incubations conducted? How would seasonal variability in assemblage structure, light or productivity affect the patterns? Are the results representative of the annual cycle? It would be useful to include a statement about the time of sampling and possible caveats of seasonal variability.

We have clarified this to state “All cBITs deployments were done in the summer (i.e. July) of each year, in similar depths (i.e. 6-8 m), and under similar wave exposures (i.e. protected from ocean swells) in order to standardize factors that could affect productivity measurements.”

Line 321: Typo with ‘PERMNOVA’

This has been deleted from the manuscript.
